# Physiological Doses of Hydroxytyrosol Modulate Gene Expression in Skeletal Muscle of Exercised Rats

**DOI:** 10.3390/life11121393

**Published:** 2021-12-12

**Authors:** Rafael A. Casuso, Saad Al Fazazi, Julio Plaza-Díaz, Francisco J. Ruiz-Ojeda, Ascensión Rueda-Robles, Jerónimo Aragón-Vela, Jesús R. Huertas

**Affiliations:** 1 Department of Physiology, Campus University of Granada, 18071 Granada, Spain; saad.alfazazi@gmail.com (S.A.F.); jeroav@ugr.es (J.A.-V.); 2 Center of Biomedical Research, Institute of Nutrition and Food Technology “José Mataix”, University of Granada, 18071 Granada, Spain; fjrojeda@gmail.com (F.J.R.-O.); ruedarobles@hotmail.com (A.R.-R.); 3 Department of Biochemistry and Molecular Biology II, School of Pharmacy, University of Granada, 18071 Granada, Spain; jrplaza@ugr.es; 4 Instituto de Investigación Biosanitaria IBS.GRANADA, Complejo Hospitalario Universitario de Granada, 18014 Granada, Spain; 5 Children’s Hospital of Eastern Ontario Research Institute, Ottawa, ON K1H 8L1, Canada; 6 RG Adipocytes and Metabolism, Helmholtz Diabetes Center at Helmholtz Center Munich, Institute for Diabetes and Obesity, Neuherberg, 85764 Munich, Germany

**Keywords:** polyphenols, mitochondria, glycolysis, gene expression

## Abstract

We tested whether physiological doses of hydroxytyrosol (HT) may alter the mRNA transcription of key metabolic genes in exercised skeletal muscle. Two groups of exercise-trained Wistar rats, HTlow and HTmid, were supplemented with 0.31 and 4.61 mg/kg/d of HT, respectively, for 10 weeks. Another two groups of rats were not supplemented with HT; one remained sedentary and the other one was exercised. After the experimental period, the soleus muscle was removed for qRT-PCR and western blot analysis. The consumption of 4.61 mg/kg/d of HT during exercise increased the mRNA expression of important metabolic proteins. Specifically, 4.61 mg/kg/d of HT may upregulate long-chain fatty acid oxidation, lactate, and glucose oxidation as well as mitochondrial Krebs cycle in trained skeletal muscle. However, a 4.61 mg/kg/d of HT may alter protein translation, as in spite of the increment showed by CD36 and GLUT4 at the mRNA level this was not translated to higher protein content.

## 1. Introduction

Extra virgin olive oil (EVOO) is a source of monounsaturated fatty acid widely consumed as part of the Mediterranean diet. Consumption of EVOO has been inversely associated with the development and management of a number of metabolic diseases, including cardio-vascular diseases and type 2 diabetes [1,2]. Moreover, EVOO supplementation changes the composition of the skeletal muscle mitochondrial membrane by increasing the amount of monounsaturated fatty acids while decreasing polyunsaturated fatty acid content [3] which, surprisingly, increases membrane fluidity [4]. This likely explains the fact that EVOO improves mitochondrial resistance against exercise-induced oxidative stress [5]. Accordingly, 30 days of EVOO supplementation to rats reduced the deleterious effect that highly intense exercise has on mitochondrial ultrastructure [6]. During moderate exercise intensity, EVOO has been reported to improve exercise performance [7]. It is known that during moderate exercise intensity, mitochondria play a primary role in skeletal muscle performance [8]. Consequently, EVOO supplementation may boost mitochondrial metabolism during exercise. However, the potential molecular mechanisms by which EVOO may improve skeletal muscle and mitochondrial function is unclear.

For instance, polyphenols from olive oil are known to have antioxidant and anti-inflammatory systemic properties [9,10]. In fact, high concentrations of hydroxytyrosol (HT) ranging from 10 to 50 mg/kg/d can alter skeletal muscle metabolism [11,12,13,14]. Surprisingly, we have recently reported that lower HT doses (i.e., 0.31 to 4.61 mg/kg/d) alter the activity of the molecular regulators of skeletal muscle glucose uptake in trained rats [15]. However, whether this alteration is specific to the glucose metabolism pathway and whether it also compromises gene transcription is yet to be determined. Therefore, we tested whether physiological doses of HT can induce the mRNA expression of key metabolic genes regulating mitochondrial fuel oxidation.

## 2. Materials and Methods

### 2.1. Animals

Six-week-old male Wistar rats were purchased from Charles River (USA). The rats initially weighed 200 ± 15.8 g and were maintained in a well-ventilated room. This room was maintained under standard conditions of temperature (21 ± 2 °C) and relative humidity (40–60%) and under a reverse 12 h light/12 h dark cycle. All rats were allowed ad libitum access to water and standard chow (2.9 kcal/g) throughout the experimental period. Daily food and water intakes were monitored. All interventions lasted for 10 weeks. Rats were randomly allocated into a sedentary group (SED, n = 6) or one of the exercised groups for 10 weeks. There were three exercised group: EXE (n = 6), HT low (n = 6) and HT mid (n = 6) (see descriptions below). Exercise was divided into 2 mesocycles of 5 weeks; each mesocycle increased the running volume from 20 min up to 65 min at 75% of the animal’s maximal running velocity as previously described in detail [16]. A total of 72 h after the last exercise was performed, the rats were fasted overnight, anesthetized with pentobarbital, and killed by bleeding. The soleus muscle was collected for analysis.

### 2.2. Hydroxytyrosol Treatment

Hydroxytyrosol is a type of phenolic compound characterized by a phenethyl alcohol structure with a molecular weight of 154.16. According to the PREDIMED study, an average person from the Spanish population consumes 30 to 50 g of EVOO daily [17], resulting in a consumption of ~22 mg of polyphenols derived from EVOO daily [17,18]. For a person of 70 kg, this would be a dose of 0.31 mg/kg/d. Therefore, the low HT dose (HTlow) was set at 0.31 mg/kg/d. However, most studies on HT use doses are above 10 mg/kg/d (see above). Therefore, we chose to explore a moderate dose, which was set at 4.6 mg/kg/d (HTmid). To achieve this dose, an ergogenic supplementation would be needed as it corresponds to a daily intake of 0.6 L of EVOO [17]. These HT doses are known to alter the molecular regulators of glucose uptake within exercised skeletal muscle [15]. HT was diluted in water in an opaque drinking bottle to prevent oxidation. The dilution was adjusted weekly according to the weight of each rat and its average water intake. This procedure is reliable for HT supplementation [13]. Supplementation stopped 12 h before rats were euthanized. The exercised control groups (EXE) were not supplemented with HT.

### 2.3. Quantitative Real-Time (qRT)-PCR

We used the RealTime ready Custom Panel 96 (Roche, Barcelona, Spain), which is a two-step qRT-PCR platform. Briefly, total ribonucleic acid (RNA) was extracted from the soleus muscle using the PeqGOLD HP Total RNA kit (Peqlab, Germany), according to the manufacturer’s recommendations. Isolated RNA was then treated with Turbo DNase (Ambion, Life Technologies, Carlsbad, CA, USA). Final RNA concentration and quality were determined using a NanoDrop 2000 (NanoDrop Technologies, Winooski, VT, USA). Complementary DNA (cDNA) was synthesized from total RNA using the iScript advanced cDNA Synthesis Kit (Bio-Rad Laboratories, Hercules, CA, USA). The RealTime ready Custom Panel 96 (Roche, Barcelona, Spain) included the following specific primer pairs: Slc2a4 (Assay ID 500810, Roche, Barcelona, Spain), Slc16a1, (Assay ID 506515, Roche, Barcelona, Spain), Cd36, (Assay ID 506518, Roche, Barcelona, Spain), Pfkm, (Assay ID 506511, Roche, Barcelona, Spain), Idh3a (Isocitrate dehydrogenase [NAD] subunit alpha, mitochondrial Precursor (EC 1.1.1.41) (Isocitric dehydrogenase subunit alpha) (NAD(+)-specific ICDH subunit alpha) [Source: UniProt KB/Swiss-Prot; Acc: Q99NA5]), Idh2 (Assay ID 506513, Roche, Barcelona, Spain). The Rat 18S rRNA sequence (Assay ID 502300, Roche, Barcelona, Spain) and hydroxymethylbilane synthase (Hmbs) (Assay ID 502305, Roche, Barcelona, Spain) were used as reference genes. The cDNA was then subjected to qRT-PCR analysis with the LightCycler^®^ 480 Probes Master Kit (Roche, Barcelona, Spain) on a LightCycler^®^ 480 Instrument II detector (Roche, Barcelona, Spain). The PCR conditions were 1 cycle at 95 °C for 10 min, followed by 45 cycles at 95 °C for 10 s, 60 °C for 30 s, and 72 °C for 1 s, and 1 cycle at 40 °C for 30 s. The expression level of each gene was analyzed with RT2 Profiler PCR Array Data Analysis software (version 3.4, SABiosciences). Changes in gene expression were expressed as fold changes (Fc).

### 2.4. Western Blotting

Soleus muscle samples were harvested in 10 mM Tris-HCl (pH 7.5), 150 mM NaCl, 2 mM EDTA, 1% Triton X-100, 10% glycerol, and a protease inhibitor cocktail (Thermo Scientific), and were then placed on ice for 20 min. After centrifugation (30 min, 13,000× *g*, 4 °C), the protein content in the supernatant was measured using a Protein Assay Kit II (Bio-Rad Laboratories, California, USA). Samples containing 30 µg of protein were mixed with 3X SDS-PAGE sample buffer (100 mM Tris-HCl, pH 6.8, 25% SDS, 0.4% bromophenol blue, 10% β-mercaptoethanol, and 2% glycerol), separated via SDS-PAGE using TGX Any kD gel (Bio-Rad Laboratories, California, USA), and transferred onto a nitrocellulose membrane (Bio-Rad Laboratories, California, USA).

After incubation in blocking buffer (5% non-fat milk and 1% Tween 20 in Tris-buffered saline, TBS), membranes were probed with the following antibodies: rabbit anti-CD36 antibody (dilution 1:1000 in 5% bovine serum albumin) acquired from Ther-moFisher Scientific (Waltham, WA, USA, PA1-16813), anti-GLUT4 (sc-53566; 1:100 in 5% non-fat milk) and anti-Hsp-70 (internal control; 1:500 in 5% non-fat milk) acquired from Santa Cruz Biotechnology (Santa Cruz, CA, USA, sc-7298). Immunoreactive signals were detected via enhanced chemiluminescence (SuperSignal West Dura Chemi-luminescent Substrate, 34075, Thermo Scientific, Europe) and membranes were digitally imaged and quantified by densitometry using ImageJ software. Results are represented as fold-change (Fc) in expression relative to the control.

### 2.5. Statistical Analysis

Results are shown as the mean ± SD. Homoscedasticity and normality were tested by Levene’s test and the Kolmogorov-Smirnov test, respectively. One-way ANOVA was used for data analysis. A post hoc analysis was performed, and confidence intervals were adjusted using the Bonferroni correction when the effect was significant. The level of significance was set at *p* < 0.05. All analyses were performed using the Statistical Package for Social Sciences (SPSS, version 22 for Windows; IBM Corp., Armonk, NY, USA).

## 3. Results

### 3.1. mRNA Expression in Soleus Muscles

**Figure 1:**
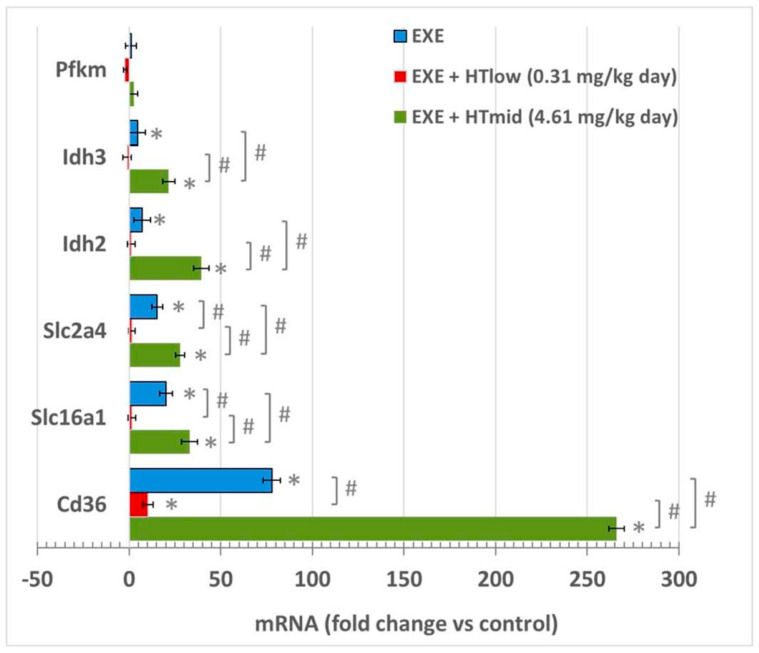
Relative mRNA levels of key metabolic pathways. Data are shown as fold change compared to control (sedentary) animals and as mean ± SD. EXE, exercised animals; HT, hydroxytyrosol; Pfkm, phosphofructokinase; Idh, isocitrate dehydrogenase; Slc2a4; glucose transporter 4; Slc16a1; monocarboxylate transporter 1; Cd36; fatty acid translocase/CD36. * *p* < 0.05 compared to sedentary animals; # *p* < 0.05.

First, we analyzed the mRNA level of the selected glycolytic genes in the soleus skeletal muscle. EXE animals showed an increased expression of the glucose transporter 4 (GLUT 4) and the monocarboxylate transporter 1 (MCT1) when compared with sedentary animals (Figure 1). Notably, this effect was magnified in HTmid animals, but absent in the HTlow group (Figure 1). None of the experimental groups showed an increase in the main regulatory enzyme of glycolysis, that is, phosphofructokinase (PFK).

We next assessed the expression of isocitrate dehydrogenase (IDH), which is an enzyme belonging to the Krebs cycle. This enzyme catalyzes the conversion of isocitrate to α-ketoglutarate (Figure 1). Notably, it has been reported that IDH2 isoform is mainly present in oxidative fibers, while IDH3 isoform is found in glycolytic fibers [19]. Both IDH2 and IDH3 mRNA levels were increased in the skeletal muscle of EXE animals (Figure 1). However, HTmid animals showed a 40-fold expression of IDH2 (*p* < 0.001) and a 25-fold expression of IDH3, which were higher than their expression in EXE and HTlow groups.

Next, we investigated the mRNA levels of the fatty acid transporter fatty acid translocase (CD36) (Figure 1). Gene expression of CD36 was induced in all the experimental groups. However, HTmid group showed a higher expression of CD36 than the EXE and HTlow groups.

### 3.2. CD36 and GLUT4 Protein Content

**Figure 2:**
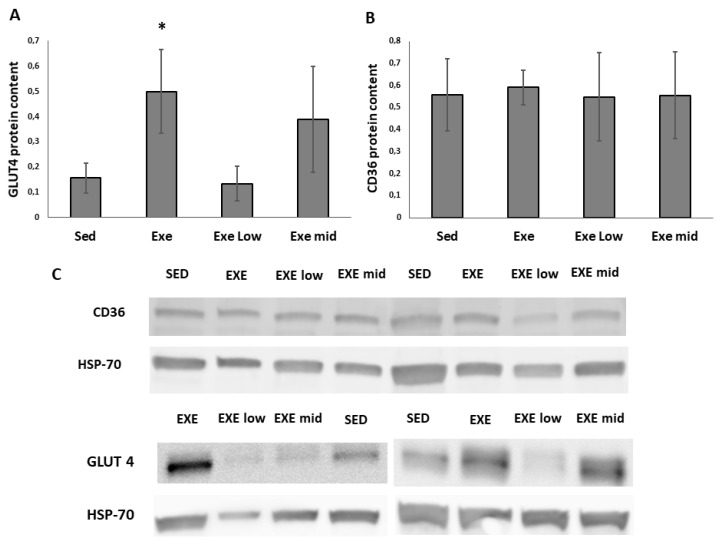
GLUT4 (**A**) and CD36 (**B**) protein content. Panel (**C**) shows representative images of CD36 and GLUT4. SED, sedentary; EXE, exercised; EXElow, exercised and supplemented with 0.31 mg/kg/d of hydroxytyrosol; EXEmid, exercised and supplemented with 4.61 mg/kg/d. Note that the GLUT4 images shown in panel (**C**) are from two membranes; in the first one the experimental group does not follow the same order as in the other membranes. * *p* < 0.05 if compared with Sed.

Surprisingly, the high mRNA levels of CD36 were not translated into an efficient protein expression (Figure 2; Supplementary material). In fact, western blot analysis showed similar CD36 protein levels between experimental groups (Figure 2B). To exclude the possibility that the blunted translation was related to the fatty acid oxidation pathway, we also assessed glucose transporter GLUT4. We found that in animals supplemented with 4.61 mg/kg/d of HT, GLUT4 protein content was highly variable (Figure 2A).

## 4. Discussion

In the present study, we show that HT supplementation at physiological doses during exercise can upregulate the expression of key metabolic proteins. Notably, this effect seems to be dose-dependent because we found that 4.61 mg/kg/d HT induces higher mRNA expression of metabolic genes than 0.31 mg/kg/d HT. To date, the in vivo effects of HT have been mainly tested in the context of the antioxidant effects induced by dosages ranging from 10–50 mg/kg/d [11]. In addition, HT inducing effects on skeletal muscle metabolism have also been tested in response to ~20 mg/kg/d of HT [11,13,14]. Our data demonstrate that the medium physiological dose of HT (4.6 mg/kg/d) can increase the expression of key metabolic genes in the skeletal muscle of trained rats.

In fact, we show that lower HT doses increase the gene expression of various proteins relevant for exercise metabolism. For instance, glucose oxidation is important due to its contribution to both anaerobic and aerobic glycolysis [9]. In this regard, GLUT4 is a transporter facilitating glucose uptake from the blood and its delivery into the cytosol of the skeletal muscle cell [20]. In addition, the monocarboxylate transporter MCT1 is responsible for the lactate uptake from the circulation for oxidation [21,22,23]. Another key metabolic transporter is CD36, which can deliver circulating long-chain fatty acids into the mitochondria for B-oxidation [24,25]. Finally, we also determined IDH gene expression as a marker of Krebs cycle function. It is noteworthy that IDH2 is mainly expressed in oxidative type I fibers, whereas IDH3 is mainly expressed in glycolytic fibers [19,26]. We found a higher expression of IDH2 and a moderate, but significant, expression of IDH3 induced by exercise.

This result is in line with the phenotype of the studied muscle, which is considered a red or oxidative muscle due to its high composition of type I oxidative fibers. Moreover, a similar expression pattern but magnified was also found in animals consuming 4.6 mg/kg/d of HT compared with EXE animals. In conjunction, our results suggest that physiological doses of HT potentiate the exercise-induced mRNA expression of key metabolic proteins. However, several differences were found between the two doses tested. While 0.31 mg/kg/d only modulates fatty acid oxidation as evidenced by an increase in mRNA levels of CD36, a dosage of 4.6 mg/kg/d may enhance the metabolic pathways related to long-chain fatty acid oxidation, lactate, and glucose oxidation, as well as mitochondrial Krebs cycle. However, the mechanisms by which HT induces mRNA expression are mostly unknown.

Recently, the biological effects of HT have been tested beyond its antioxidant and anti-inflammatory effects. Indeed, a relatively new research topic aims to identify the epigenetic effects that HT can induce on the DNA promoters of key regulatory proteins. For instance, it has been observed that HT can rescue oxidative stress-induced DNA hypomethylation in the offspring of female mice suffering from intrauterine growth restriction [27]. Moreover, 50 μM of HT increases the expression of the anticancer gene type 1 cannabinoid receptor (CB1) by decreasing the methylation of its DNA promoter site [28]. Notably, significantly lower doses of HT, ranging from 0.1–10 μM, can regulate mitochondrial metabolism by activating PGC-1α promoter activity [29]. Although the latter study did not measure epigenetic changes in the PGC-1α promoter, these results are consistent with recent findings reporting that EVOO supplementation can modulate metabolism by changing the methylation levels of genes related to metabolism in peripheral white blood cells [30]. These studies are in the context of clinical diseases; however, these data collectively suggest that HT can regulate gene expression through modulating the methylation levels of the DNA promoter. These results are noteworthy because they contribute to the study of phytochemicals from another point of view, which may help to gain insight into their biological and medicinal properties.

It is important to note that the doses applied in the present study prevent the exercise-induced rise in the skeletal muscle glucose uptake pathway. Indeed, both RAC1 and AKT activities are blunted when 4.6 mg/kg/d of HT is supplemented during exercise [15]. Similarly, here we show that while CD36 mRNA levels are higher in the group consuming 4.6 mg/kg/d of HT (HTmid) if compared to the other experimental groups, this is not translated into higher CD36 protein content. Moreover, GLUT4 protein content showed a high variability pattern in the animals supplemented with 4.61 mg/kg/d. Therefore, suggesting that while HT is able to increase transcripts levels of key metabolic enzymes within the trained muscle, this is not properly translated into functional proteins. It should however be noted that there are several glucose carriers within skeletal muscle and some of them have unknown substrates and function like GLUT11 [31]. Thus, future studies on the effects of polyphenols in muscle glucose uptake would help to characterize the function of less studied glucose carriers.

It is important to highlight that HT can increase the amount of mitochondrial respiratory complexes into supercomplexes by a yet uncharacterized mechanism [14,32]. Our observation that HT prevents GLUT4 protein expression could provide a mechanistic link. In fact, glucose starvation within endoplasmic reticulum (ER) activates the unfolded protein response which through the PERK-eIF2a-ATF4 axis stimulates the assembly of mitochondrial supercomplexes [33]. In addition, the PERK arm of the unfolded protein response is a crucial checkpoint in stress-dependent proteostasis responses [34] as it stops global protein synthesis by the inhibition of protein translation through eIF2a and ATF4 [35]. This mechanism mimics our results, showing an increase in mRNA transcription but not protein expression. However, further work should elucidate whether the lack in protein concentration expression induced by HT can be explained by the induction of ER stress through limiting the amount of cellular glucose availability and/or by another mechanism by which HT could induce the PERK-eIF2a-ATF4 axis. In addition, it will be also important to study if HT induces autophagy (which is also activated by ATF4), or if it activates miRNAs which could also lead to the observed lack of modification in protein content.

## 5. Conclusions

The consumption of 4.61 mg/kg/d of HT can boost the mRNA levels of key metabolic proteins induced by exercise. The molecular mechanisms triggered by HT inducing such effects are unknown yet. Nevertheless, future studies must test the epigenetic modifications induced by HT at physiological doses and/or by EVOO supplementation. It must be noted that HT consumption at physiological doses during training might impede the translation of key mRNA transcripts within skeletal muscle.

## Data Availability

Data is available from corresponding authors upon reasonable request.

## Supplementary Materials

The following are available online at https://www.mdpi.com/article/10.3390/life11121393/s1, Figure S1: Relative to Figure 2. CD36 gel, Figure S2: Relative to Figure 2. HSP70 from CD36 gel, Figure S3: Relative to Figure 2. Glut4 gel, Figure S4: Relative to Figure 2. HSP70 from GLUT4 gel.
