# Supplementary material for: Physiological Doses of Hydroxytyrosol Modulate Gene Expression in Skeletal Muscle of Exercised Rats
Source: Life (Basel). 2021 Dec 12;11(12):1393. doi: 10.3390/life11121393 (PMC8708182; doi:10.3390/life11121393)
Supplement: Supplementary file 1 [file life-11-01393-s001.zip › life-1489799-supplementary.pdf]

Physiological Doses of Hydroxytyrosol Modulate Gene Expression in Skeletal Muscle of Exercised Rats

Rafael A. Casuso, SaadAl Fazazi, Julio Plaza-Díaz, Francisco Javier Ruiz-Ojeda, Ascensión Rueda-Robles, Jerónimo Aragón-Vela, and Jesús R. Huertas

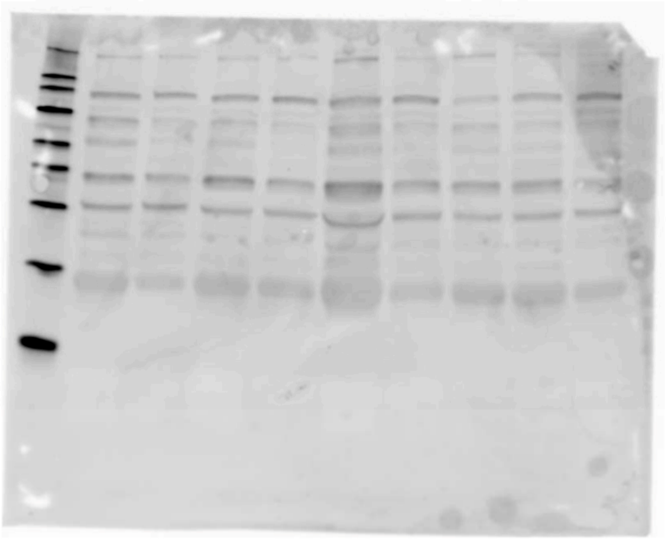

Supplementary Figure S1. Relative to Figure 2.
CD36 gel

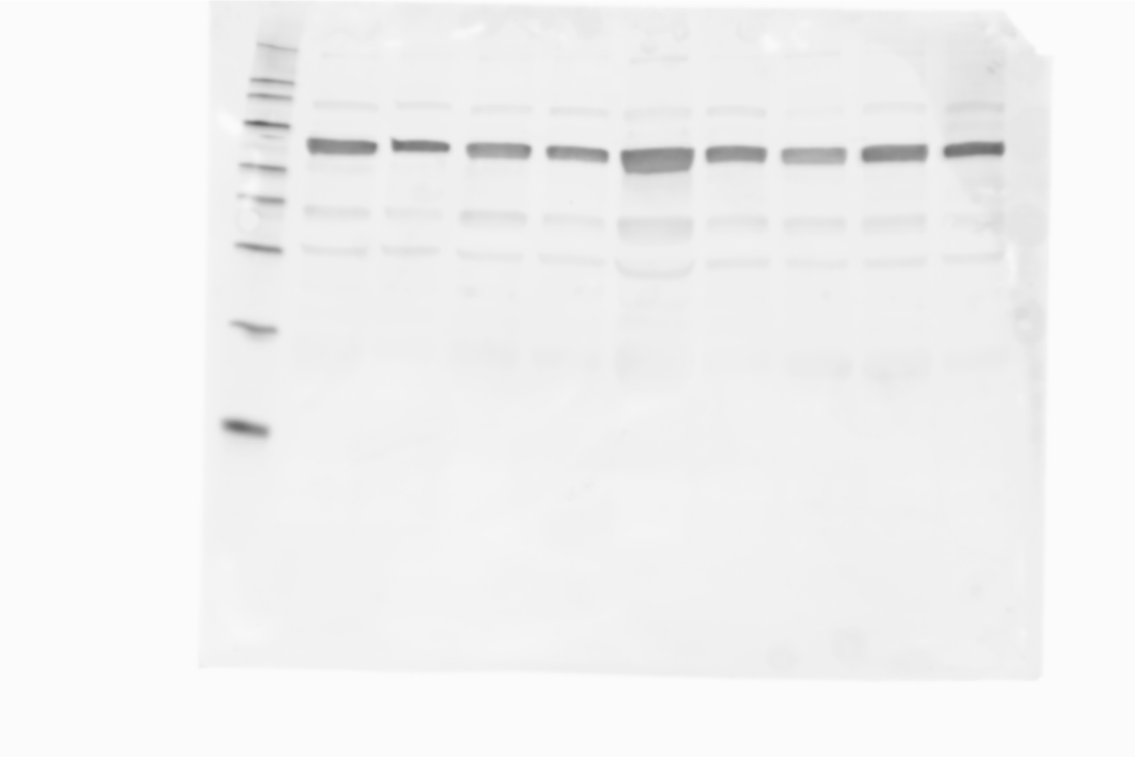

Supplementary Figure S2. Relative to Figure 2.
HSP70 from CD36 gel

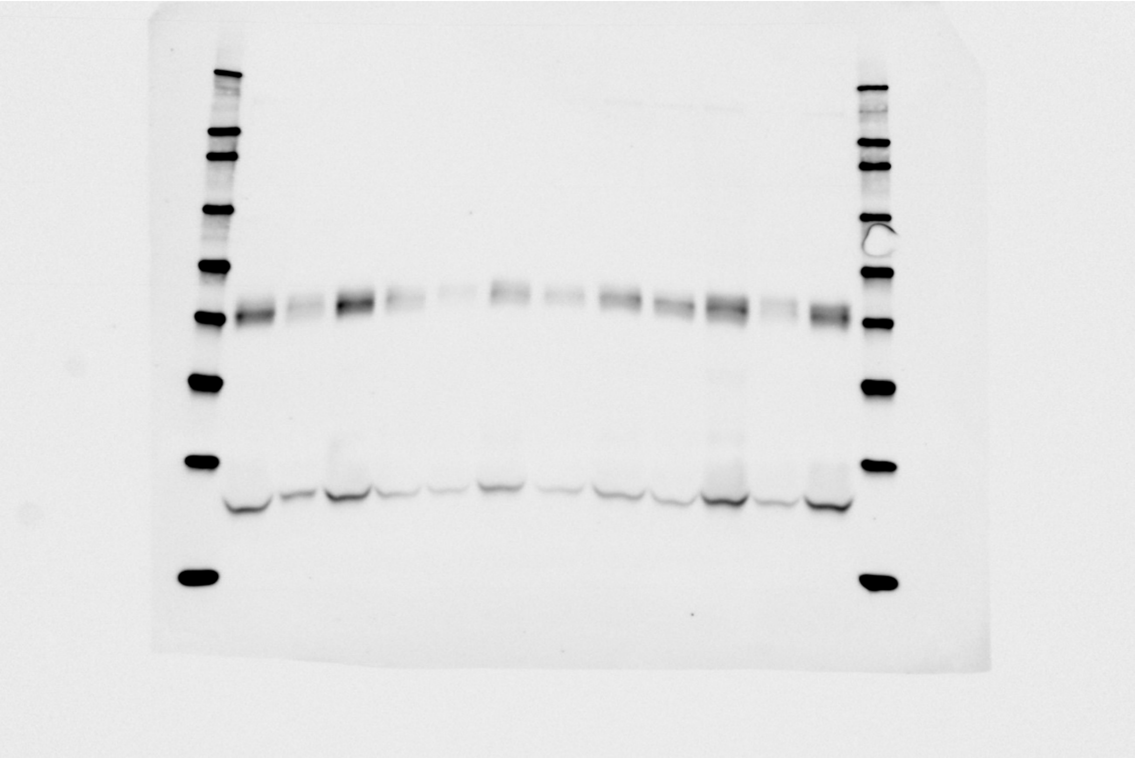

Supplementary Figure S3. Relative to Figure 2.
Glut4 gel

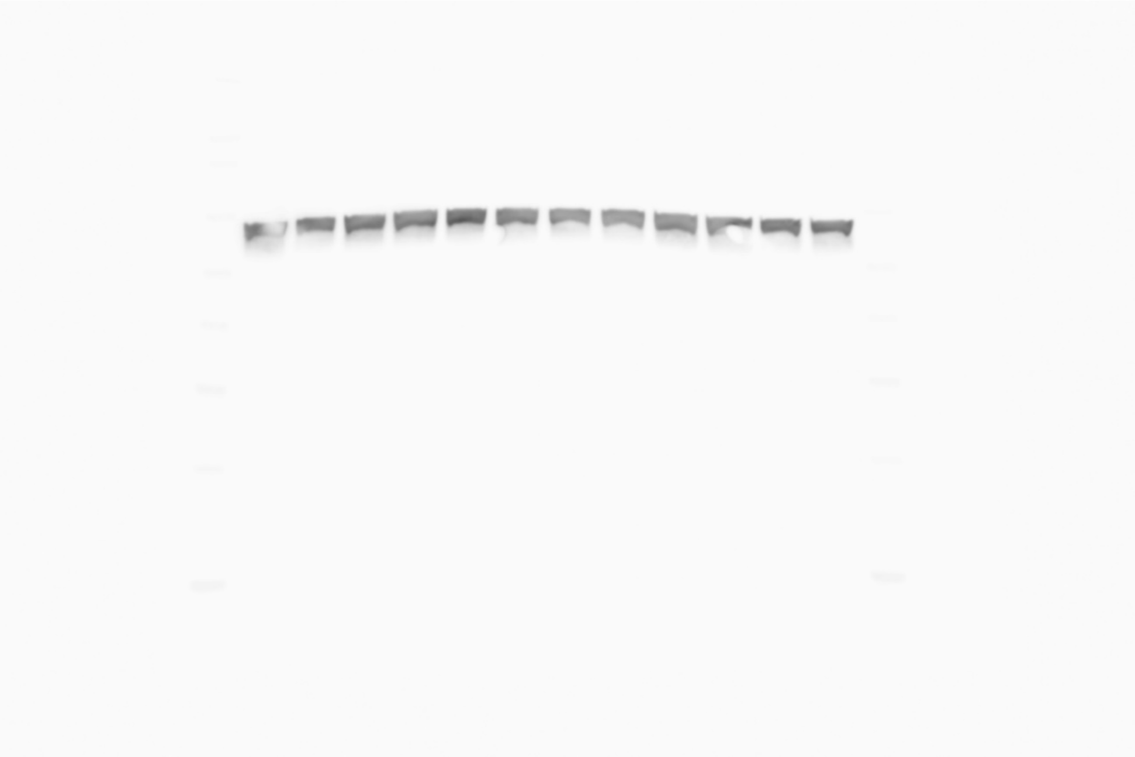

Supplementary Figure S4. Relative to Figure 2.
HSP70 from GLUT4 gel
